# Silver-functionalized carbon nanofiber composite electrodes for ibuprofen detection

**DOI:** 10.1186/1556-276X-7-331

**Published:** 2012-06-21

**Authors:** Florica Manea, Sorina Motoc, Aniela Pop, Adriana Remes, Joop Schoonman

**Affiliations:** 1 Politehnica” University of Timisoara, Timisoara, 300006, Romania; 2 Department ChemE, Delft University of Technology, Delft, 2600 AA, The Netherlands

**Keywords:** Carbon nanofiber composite electrodes, Silver particles, Electrochemical determination, Ibuprofen

## Abstract

The aim of this study is to prepare and characterize two types of silver-functionalized carbon nanofiber (CNF) composite electrodes, i.e., silver-decorated CNF-epoxy and silver-modified natural zeolite-CNF-epoxy composite electrodes suitable for ibuprofen detection in aqueous solution. Ag carbon nanotube composite electrode exhibited the best electroanalytical parameters through applying preconcentration/differential-pulsed voltammetry scheme.

## **Background**

Nanoscale carbonaceous materials, especially carbon nanotubes (CNTs) and carbon nanofibers (CNFs), have attracted great research interests in the electroanalysis field. The development of carbon nanofiber-based composite electrodes combine the enhanced electrical properties and ease of processing of such electrodes exhibiting attractive electrochemical and economical features [1,2]. However, the improvement of the electroanalytical signal requires catalyst incorporation into the composite matrix, and silver-decorated CNT has been reported [3]. In recent years, there has been increasing concern about the presence of pharmaceutical compounds in water, known as emerging pollutants. Ibuprofen (IBP) is the third most popular drug in the world, and its presence in water requires viable methods for its determination. Several determination methods have been reported in the literature e.g., spectrophotometry [4,5], HPLC [6], and an electrochemical method [7]. To the best of our knowledge, there is no information about ibuprofen detection using a nanostructured carbon-based electrode. In this paper, two types of silver-functionalized CNF composite electrodes, silver-decorated CNF-epoxy (AgCNF) and silver-modified natural zeolite-CNF-epoxy (AgZCNF) composite electrodes were prepared, morphologically characterized, and applied for IBP detection in aqueous solution.

## **Methods**

CNFs with diameters of 60 to 150 nm and lengths of 30 to 100 μm were purchased from Applied Sciences Inc., Cedarville, Ohio (Pyrograf III - PR24 AGLD). Silver-modified zeolite was prepared by ion-exchange using natural zeolite (NZ) from Mirsid, Romania (68 wt.%;) as we previously described [8]. Araldite®LY5052/Aradur®5052 two-component epoxy resin used in the study was purchased from Huntsman Advanced Materials, Basel, Switzerland. The decoration of the CNF composite with silver nanoparticles was carried out by reducing silver ions in the presence of *NN*-dimethylformamide (DMF). A 1.1-g CNF was added to 550 mL of DMF, and the mixture was subjected to ultrasonication (Cole-Parmer 8900, Vernon Hills, Chicago, IL, USA) for 1 h; 40 mL of an AgNO_3_ solution (0.02 M) was added into the mixture with the temperature of 60°C to 62°C during the stirring. After 1 h, heating the solution was kept without stirring at room temperature for 48 h for Ag deposition, and after filtration and subsequently washing with water, ethanol, and acetone, silver-decorated CNF was obtained. The composite electrodes were prepared by dispersion of CNFs in DMF, 99.9%; (DMF, Sigma-Aldrich, Corporation, St. Louis, MO, USA) and epoxy resin (Araldite®LY5052) by ultrasonication, followed by the homogenization of the resulting paste with the zeolite particles and also with the hardener using a two-roll mill. The mixture was then poured into PVC tubes and cured at 60°C for 24 h obtaining disc electrodes with a surface area of 0.196 cm^2^. The ratios were chosen to obtain 20 wt.%; CNFs for the AgCNF electrode and also 20 wt.%; Ag-modified zeolite for the AgZCNF electrode. Scanning electron microscopy coupled with energy dispersive X-ray analysis detection (SEM/EDX) was performed using an Inspect S PANalytical instrument (PANalytical B.V., Almelo, The Netherlands). Electrochemical measurements were carried out using an Autolab PGSTAT101 (Metrohm Autolab, Utrecht, The Netherlands) controlled with the NOVA 1.6 software and a three-electrode cell with an Ag/AgCl reference electrode, a platinum counter electrode, and the composite working electrodes. Cyclic voltammetry (CV), differential-pulsed voltammetry (DPV), and chronoamperometry (CA) were used to assess the electroanalytical performance of both composite electrodes for IBP detection in the aqueous solution.

## **Results and discussion**

### **Surface characterization**

**Figure 1:**
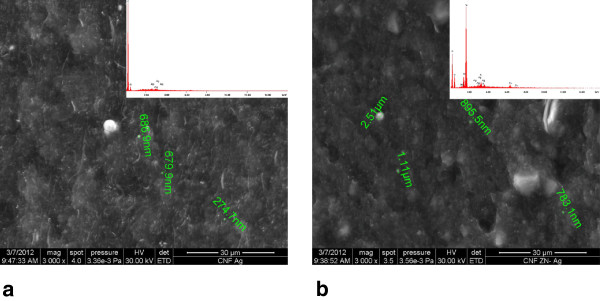
SEM/EDX images of (a) AgCNF and (b) AgZCNF electrode materials.

Figure 1a,b presents the SEM/EDX images of both electrodes, i.e., AgCNF and AgZCNF composite electrodes, and a homogeneous distribution of CNF within the epoxy matrix is observed. Higher content of Ag particles was found for silver-modified natural zeolite-CNF-epoxy in comparison with silver-decorated CNF-epoxy composite materials.

### **Cyclic voltammetric measurements**

**Figure 2:**
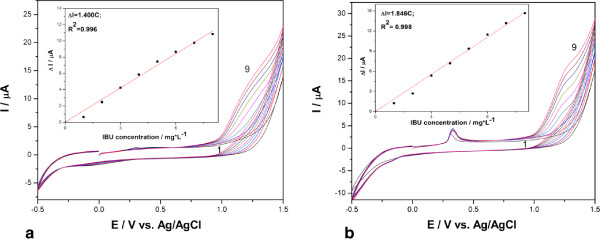
**Recorded cyclic voltammograms.** These were recorded at (**a**) AgCNF, (**b**) AgZCNF electrodes in 0.1 M Na_2_SO_4_ supporting electrolyte (curve 1) and in the presence of various IBP concentrations that consists 1 to 8 mg.L^−1^ (curves 2 to 9); potential scan rate of 50 mV s^−1^. Inset: calibration plots of peak current vs. IBP concentration.

Figure 2a,b shows the series of the cyclic voltammograms recorded at AgCNF and AgZCNF composite electrodes in 0.1 M Na_2_SO_4_ supporting electrolyte and in the presence of various IBP concentrations. Similar shapes of the voltammograms can be noticed for both electrodes, and the oxidation peak corresponding to the Ag/Ag ^+^ couple appeared at the potential value of +0.3 V/SCE, which is more evidenced for AgZCNF in direct relation with the silver content. The current corresponding to the IBP oxidation peak recorded at about +1.3 V vs. Ag/AgCl increased progressively with its concentration (see insets of Figure 2). On the following reverse scan from 1.5 to −0.5 V vs. Ag/AgCl, no corresponding reduction peak is observed, revealing that the electrode process at both electrodes is totally irreversible.

### **Electrochemical detection of ibuprofen**

The simplest electroanalytical procedure should involve the recording of the chronoamperogram, based on the reference provided by the cyclic voltammograms. Thus, for both electrodes, the continuous chronoamperograms were recorded at the potential value of + 1.3 V vs. Ag/AgCl within the IBP concentration range between 0.5 and 5 mg.L^−1^. The sensitivity was compared to CV for AgCNF, while for AgZCNF, a lower sensitivity was achieved probably due to electrode fouling that occurred under these working conditions (see Table 1).

**Table 1:** **Electroanalytical performances of AgCNF and AgZCNF composite electrodes for IBP detection in 0.1-M Na**_**2**_**SO**_**4 **_**supporting electrolyte**

**Used technique**	**Electrode material**	**E/V**	**Electrode sensitivity(μA/mg.L**^**−1**^**)**	**Correlation coefficient (R2)**	**Concentration range(mg.L**^**−1**^**)**	**RSD(%;)**	**LOD (mg.L**^**-1**^**)**
CV	AgZCNF	+1.29	1.846	0.998	1 to 8	3.50	0.03
	AgCNF	+1.3	1.400	0.996	1 to 8	3.20	0.02
DPV	AgZCNF	+1.17	2.867	0.993	0.5 to 5	3.75	0.008
	AgCNF	+1.15	1.810	0.977	0.5 to 5	3.90	0.01
Preconcentration/DPV	AgCNF	+1	4.150	0.998	0.5 to 5	4.25	0.001
SWV	AgZCNF	+1.25	2.27	0.988	0.5 to 5	4.20	0.009
	AgCNF	+1.2	2.140	0.992	0.5 to 5	3.80	0.008
CA	AgZCNF	+1.3	0.317	0.975	0.5 to 4.5	1.40	0.03
	AgCNF	+1.3	1.636	0.944	0.5 to 4.5	1.25	0.02

E/V, detection potential/Volt vs. Ag/AgCl; LOD, lowest limit of detection; RSD, relative standard deviation; SWV, square-wave voltammetry.

**Figure 3:**
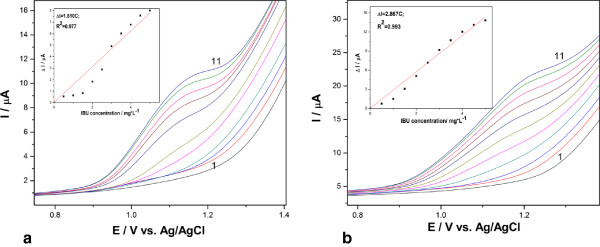
**Recorded differential-pulsed voltammograms (0.2 V modulation amplitude, 0.02 V step potential).** They were recorded at (**a**) AgCNF, (**b**) AgZCNF electrodes in 0.1 M Na_2_SO_4_ supporting electrolyte and in the presence of various IBP concentrations that consists 0.5 to 5 mg.L^−1^ (curves 2 to 11). Inset: calibration plots of the anodic current recorded at E = +1.15 V vs. IBP concentration.

In order to improve the electroanalytical performances of both electrodes for IBP detection, the DPV technique was applied after optimization of its parameters. Figure 3a,b presents DPVs recorded at both electrodes, and the sensitivity was improved only for the AgZCNF composite electrode. This aspect can be explained by the higher value of the background current for AgZCNF in comparison with AgCNF, which indicated a smaller amount of silver particles in AgCNF which is in according with the SEM/EDX results.

To explore the sorption capacity of both electrodes towards IBP, a preconcentration-voltammetric detection scheme was tested after the optimum accumulation time settling based on the results of the effect of accumulation time on the currents of the differential-pulse anodic peak recorded at +1.15 V vs. Ag/AgCl corresponding to IBP oxidation. The accumulation time is considered the time maintaining at open-circuit potential before running the DPV. The enhancement factor was determined as ratio of the peak current recorded at different accumulation times to that recorded without a preconcentration scheme, and the maximum value of about 4 was reached for AgCNF and about 2 for AgZCNF at the accumulation time of 20 min. At longer accumulation times, no higher current response was obtained (the results are not shown here). Applying the preconcentration-voltammetric detection scheme using the AgCNF electrode, the sensitivity for IBP detection was improved; a value of 4.15 μA/mg.L^−1^ was achieved in comparison with 1.81 μA/mg.L^−1^ without preconcentration.

## **Conclusions**

AgCNF and AgZCNF composite electrodes were successfully prepared by a two-roll mill procedure. SEM images showed the presence of silver particles and a good dispersion of CNF into the epoxy matrix. Both electrodes exhibited good sensitivities for ibuprofen determination using CV, DPV, and CA techniques. Moreover, using CA as the simplest electrochemical technique with real practical potential, a very good electroanalytical performance for IBP detection at 1.3 V vs. Ag/AgCl was reached using the AgCNF electrode. Also, the AgCNF composite electrode exhibited useful properties for applying the preconcentration-voltammetric detection technique, which allowed the achievement of a better sensitivity without electrode fouling occurring. In comparison with our previous reported work [7], better results were achieved in this study for the lowest limit of detection of ibuprofen in the aqueous solution.

## Abbreviations

AgCNF: Silver-decorated CNF-epoxy electrode; AgZCNF: Silver-modified natural zeolite-CNF-epoxy electrode; CA: Chronoamperometry; CNF: Carbon nanofibers; CNT: Carbon nanotubes; CV: Cyclic voltammetry; DMF: N:N-dimethylformamide; DPV: Differential-pulsed voltammetry; IBP: Ibuprofen; NZ: Natural zeolite; SEM: Scanning electron microscopy.

## **Competing interests**

The authors declare that they have no competing interests.

## **Authors’ contributions**

FM conceived and coordinated the study, and carried out the electrode preparation and data interpretation. SM carried out the electrochemical detection measurements. AP and AR performed SEM measurements and contributed to electrode preparation. JS participated in the design and coordination. All authors read and approved the final manuscript.

## **Authors’ information**

FM is an associate professor of Electrochemistry Applied for Environment Remediation and Monitoring. AP is a postdoctoral research associate. JS is a professor of Advanced Materials. SM and AR are Ph.D. students.
